# Prevalence and Associated Factors of Dyslipidemia in the Adult Chinese Population

**DOI:** 10.1371/journal.pone.0017326

**Published:** 2011-03-10

**Authors:** Shuang Wang, Liang Xu, Jost B. Jonas, Qi Sheng You, Ya Xing Wang, Hua Yang

**Affiliations:** 1 Beijing Institute of Ophthalmology, Beijing Tongren Hospital, Capital Medical University, Beijing, China; 2 Department of Ophthalmology, Medical Faculty Mannheim of the Ruprecht-Karls-University, Heidelberg, Germany; University of Tor Vergata, Italy

## Abstract

To determine the prevalence, associated factors, awareness and control of dyslipidemia in Chinese living in Greater Beijing, we measured the serum cholesterol concentration in 3251 Chinese adults (age: 45 to 89 years) as participants of the population-based Beijing Eye Study 2006. Additional information on treatment of dyslipidemia was obtained using a standard questionnaire. The mean concentrations of total, HDL cholesterol, LDL cholesterol and triglycerides were 4.92±1.01 mmol/L, 1.61±0.36 mmol/L, 2.88±0.85 mmol/L, and 1.76±1.29 mmol/L, respectively. Prevalence of dyslipidemia was 56.1±0.9%%. Presence of dyslipidemia was significantly associated with increasing age (odds ratio (OR):1.02; 95% confidence interval (CI): 1.01, 1.03), female gender (OR:1.51; 95%CI: 1.25, 1.83), urban region (OR:1.82; 95%CI: 1.30, 2.55), body mass index (OR:1.13; 95%CI: 1.10, 1.15), income (OR:1.11; 95%CI:1.02, 1.21), blood glucose concentration (OR:1.10; 95%CI:1.05, 1.16), diastolic blood pressure (OR:1.02; 95%CI: 1.01, 1.03), and smoking (OR:1.23; 1.01, 1.51). Among those who had dyslipidemia, the proportion of subjects who were aware, treated and controlled was 50.9%, 23.8%, and 39.91%, respectively. The awareness rate was associated with urban region (*P* = 0.001; OR: 6.50), body mass index (*P* = 0.001; OR:1.06), and income (*P* = 0.02; OR:1.14). The data suggest that dyslipidemia may be present in about 56% of the population aged 45+ years in Greater Beijing. Factors likely associated with dyslipidemia were higher age, female gender, urban region, higher body mass index, higher income, higher blood concentration of glucose, higher diastolic blood pressure, and smoking. In the examined study population, treatment rate was 24% with about 60% of the treated subjects still having uncontrolled dyslipidemia.

## Introduction

Dyslipidemia, a major systemic disorder, is one of the most important risk factors for cardiovascular diseases which are a major cause of morbidity and a leading contributor to mortality worldwide [1]. It also includes developing countries, and among them in particular China [1], [2]. Over the next 20 years, cardiovascular disease morbidity and mortality in China has been projected to increase both in absolute number and as a proportion of total disease burden [2]. The marked increase in cardiovascular diseases in economically developing countries has resulted from the economic growth and associated sociodemographic changes that have occurred over recent decades. During this period, the burden of illness from infectious disease has fallen. Parallel changes in lifestyle and diet have led to an increase in life expectancy and a greatly increased burden of cardiovascular disease and other chronic diseases [2]–[4]. Dyslipidemia is one of the most important modifiable risk factors for cardiovascular diseases [5]–[10]. Previous population-based studies form China [11]–[21], such as the Sino-MONICA Study from 1983 to 1993 [2], [11], [13], the International Collaborative Study of Cardiovascular Diseases in Asian (InterASIA) from 2000 to 2001 [15], and The Fourth Chinese National Nutrition and Health Survey from 2002 [14], revealed that the Chinese population as compared with Western societies previously had lower concentrations of serum lipids and a lower prevalence of dyslipidemia. The studies also showed a trend over the recent decades towards an increase in the prevalence of dyslipidemia in China, parallel to a change in the lifestyle. The changes predominantly took place in the urban areas, while the situation changed to a lesser degree in rural communities. There have been only few recent studies addressing the medically and socio-economically important question of the prevalence of dyslipidemia and its associated factors in rural and urban areas of China. We, therefore, assessed in our population-based investigation the frequency of dyslipidemia, its associated factors, and the awareness, treatment, and control of dyslipidemia in the urban and rural region of Greater Beijing.

## Results

### Demographic Parameters

The study included 2951 (90.8%) subjects (1674(56.7%) women) for whom serum lipids measurements were available. The mean age was 60.4±10.0 years (median: 60 years; range: 45–89 years). Out of the 2951individuals, 1407(47.7%) subjects (834 women) came from the rural region, and 1544 (52.3%) subjects (840women) came from the urban region. The subjects from the rural region compared with the subjects from the urban region were significantly younger (56.9±9.0 years versus 63.6±9.9 years; *P*<0.001), and had a significantly lower monthly income (399±310 Yuan versus 2177±594 Yuan; *P*<0.001) and a lower level of education (*P*<0.001). The participants of the survey 2006 compared with the non-participants were significantly younger (55.3±10.1 years versus 58.6±11.6 years; *P*<0.001), came more often from the rural region than from the urban region (1500/1751 versus 473/714; *P*<0.001), and had a higher level of education (*P* = 0.001). There were no significant differences in gender (*P* = 0.84) and reported income (1067±827 versus 1093±1052; *P* = 0.41).

### Laboratory Results

**Figure 1:**
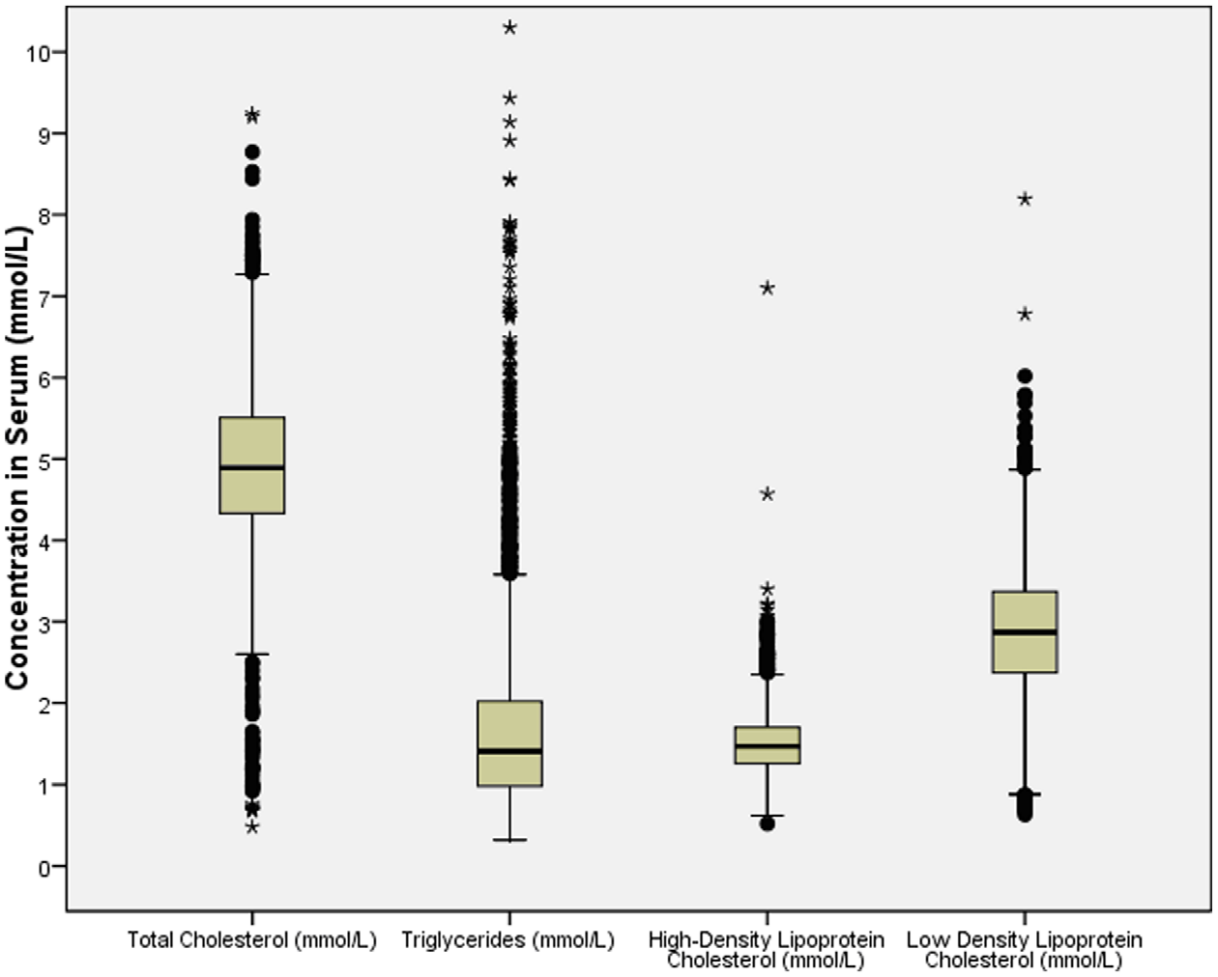
Boxplots showing the distribution of total cholesterol, triglycerides, high-density lipoprotein cholesterol and low-density lipoprotein cholesterol in the Beijing eye Study.

Mean levels of total cholesterol, HDL (high-density lipoprotein) cholesterol, LDL (low-density lipoprotein) cholesterol, and triyglcerides were 4.92±1.01 mmol / L, 1.61±0.36 mmol/L, 2.88±0.85 mmol/L, and 1.76±1.29 mmol/L, respectively (Fig. 1). A hypercholesterolemia (total cholesterol concentration ≥5.72 mmol/L (220 mg/dL)) was found in 19.0±0.7%, a hypertrigylceridemia (triglyceride concentration ≥1.70 mmol/L (150 mg/dL)) in 35.5±0.9%, and abnormally low high-density lipoprotein-cholesterol (HDL-C concentration ≤0.91 mmol/L (35 mg/dL)) in 1.7±0.2% of the study population. Dyslipidemia was found for 1332 (45.1±0.9%) subjects (95%CI: 43.3%, 46.9%). A positive history for dyslipidemia was described by 842 (28.5%) subjects, so that the total prevalence of dyslipidemia was 1654 / 2951 or 56.1±0.9% (95%CI: 54.3%, 57.8%). For all further statistical analysis, the total prevalence of dyslipidemia (abnormal blood examinations results and / or positive history for dyslipidemia) was taken. Adjusted for age and gender on the basis of Chinese census 2000, the prevalence of dyslipidemia was 54.1%.

### Univariate Analysis

In univariate analysis, all four blood laboratory parameters were significantly (*P*<0.001) correlated with each other. In univariate analysis, the prevalence of dyslipidemia was significantly associated with age, female gender, urban region, body mass index (all *P*<0.001), lower body height (*P* = 0.02), body weight (*P*<0.001), level of education (*P*<0.001), income (*P*<0.001), fasting serum concentration of glucose (*P*<0.001), systolic blood pressure (*P* = 0.02), mean arterial blood pressure (*P* = 0.03), and less consumption of wine (*P = *0.001) and less smoking (*P = *0.002) (Table 1). The prevalence of dyslipidemia was not significantly associated with consumption of beer (*P* = 0.79).

**Table 1:** Associations between the presence of dyslipidemia and other parameters in the Beijing Eye Study 2006.

Univariate analysis:			
Parameter	*P*-Value	Exponential B	95% Confidence Interval
Age	<0.001	1.02	1.02, 1.03
Female gender	0.001	1.29	1.11, 1.49
Urban region	<0.001	2.09	1.81, 2.43
Body mass index	<0.001	1.11	1.08, 1.13
Body height	0.02	0.99	0.98, 0.99
Body weight	<0.001	1.02	1.02, 1.03
Level of education	<0.001	1.15	1.08, 1.23
Income	<0.001	1.20	1.15, 1.24
Fasting serum concentration glucose concentration	<0.001	1.14	1.08, 1.20
Systolic blood pressure	0.02	1.01	1.01, 1.02
Mean arterial blood pressure	0.03	1.01	1.01, 1.02
Consumption of wine	0.001	0.86	0.78, 0.94
Smoking	0.002	0.79	0.67, 0.92
Consumption of beer	0.79		

Binary regression analysis:			
Parameter	*P*-Value	Exponential B	95% Confidence Interval
Age	<0.001	1.02	1.01, 1.03
Gender	<0.001	1.51	1.25, 1.83
Urban region	0.001	1.82	1.30, 2.55
Body mass index	<0.001	1.13	1.10, 1.15
Income	0.01	1.11	1.02, 1.21
Blood concentr. of glucose	<0.001	1.10	1.05, 1.16
Diastolic blood pressure	0.02	1.02	1.01, 1.03
Smoking	0.04	1.23	1.01, 1.51
Level of education	0.40		
Systolic blood pressure	0.56		
Consumption of wine	0.24		

### Multivariate Analysis

In a binary multivariate logistic regression analysis, with the presence of dyslipidemia as dependent variable and all parameters, which were significantly associated with dyslipidemia in univariate analysis, as independent variables, revealed that the presence of dyslipidemia was still significantly associated with increasing age (*P*<0.001), female gender (*P*<0.001), urban region (*P* = 0.001), body mass index (*P*<0.001), income (*P* = 0.01), blood concentration of glucose (*P*<0.001), diastolic blood pressure (*P* = 0.02), and smoking (*P* = 0.04) (Table 1). In the binary logistic regression analysis with adjustment for age, gender, urban versus rural region, body mass index, income, blood glucose concentration, diastolic blood pressure and smoking, the prevalence of dyslipidemia was no longer associated with level of education (*P* = 0.40), systolic blood pressure (*P* = 0.56) and consumption of wine (*P* = 0.24).

### Awareness of Dyslipidemia

Out of the 2951 study participants, 842 (28.5%) subjects (28.5%±0.8%; 95%CI: 26.9%, 30.2%) or 842 subjects out of the 1654 subjects with dyslipidemia (50.9%±1.2%; 95%CI: 48.5%, 53.3%) reported of any previous diagnosis of dyslipidemia by a healthcare professional. Within the group of subjects with dyslipidemia, the prevalence of the awareness of dyslipidemia was significantly associated (univariate analysis) with increasing age (*P*<0.001), male gender (*P* = 0.001), urban region (*P*<0.001), lower body mass index (*P* = 0.001), higher income (*P*<0.001), lower blood concentration of glucose (*P* = 0.02), lower diastolic blood pressure (*P*<0.001), and less smoking (*P*<0.001) (Table 2).

**Table 2:** Associations between the awareness of dyslipidemia and other parameters in the Beijing Eye Study 2006.

Univariate analysis:			
Age	<0.001	1.04	1.03, 1.05
Male gender	0.001	0.72	0.59, 0.88
Urban region	<0.001	10.1	7.96, 12.8
Body mass index	0.001	0.96	0.93, 0.98
Income	<0.001	1.67	1.58, 1.77
Blood conc. of glucose	0.02	0.94	0.89, 0.99
Diastolic blood pressure	<0.001	0.94	0.92, 0.95
Smoking	<0.001	0.67	0.54, 0.84
Binary regression analysis:			
Urban region	0.001	6.50	4.10, 10.3
Body mass index	0.001	1.06	1.02, 1.09
Income	0.02	1.14	1.02, 1.27
Age	0.56		
Gender	0.21		
Blood conc. Glucose	0.24		
Diastolic blood pressure	0.11		
Smoking	0.97		

In a multivariate binary logistic regression analysis, with the presence of awareness of dyslipidemia as dependent variable and all parameters, which were significantly associated with dyslipidemia in univariate analysis, as independent variables, revealed that the presence of dyslipidemia was still significantly associated with urban region (*P* = 0.001), body mass index (*P* = 0.001), and higher income (*P* = 0.02), while it was no longer significantly associated with age (*P* = 0.56), gender (*P* = 0.21), blood concentration of glucose (*P* = 0.24), diastolic blood pressure (*P* = 0.11), and smoking (*P* = 0.97) (Table 2).

### Treatment of Dyslipidemia

Out of the 1654 subjects with dyslipidemia 393 (23.8%±1.0%; 95%CI: 22.0%, 26.0%) reported to be under treatment of the disorder. Out of the 393 subjects under treatment, 236 (60.1%) still had abnormally high levels of total cholesterol or of triglycerides or abnormally low high-density lipoprotein-cholesterol concentrations.

## Discussion

Our study aimed to examine the prevalence of dyslipidemia, its associated factors, awareness, treatment, and control in the urban and rural region of Greater Beijing. It revealed that the prevalence of dyslipidemia was about 56%. Transferred onto the total population of China, this figure may imply that approximately 175 million Chinese may be affected by dyslipidemia. It shows the tremendous importance, dyslipidemia may get in the next future in China.

The results of our study agree with previous populations-based studies from China. If the results of our study were compared with the findings from the InterAsia Collaborative Study [15], the mean concentrations of total cholesterol (4.92 mmol/L or 190.2 mg/dL versus 186.1 mg/dL), HDL cholesterol (1.61 mmol/L or 62.3 mg/dL versus 51.7 mg/dL), LDL cholesterol (2.88 mmol/L or 111.4 mg/dL versus 109.5 mg/dL), and triyglcerides (1.76 mmol/L or 155.3 mg/dL versus 128.1 mg/dL) did not vary markedly between both studies [15]. Also other regional studies have previously examined serum lipid concentrations in Chinese populations [14], [23]. The PRC-USA Collaborative Study in Cardiovascular and Cardiopulmonary Epidemiology, which was performed in 1983 to 1984, reported that age-adjusted mean serum total cholesterol level was higher in urban than in rural samples and generally higher in Beijing than in Guangzhou [23]. In a repeated survey conducted in the same populations during 1993 to 1994, the mean total cholesterol level increased in Guangzhou but decreased in Beijing [14]. However, this study was conducted in an occupational population sample of convenience rather than in a representative sample of the general population. A rapid increase in total serum cholesterol level has also been observed in residents living in Shanghai, China [24]. The differences in dietary nutrient intake between north and south as well as between rural and urban China may contribute to the observed regional differences in serum lipid levels [25]. It is confirmed in our study, in which the prevalence of dyslipidemia was significantly higher in the urban region than in the rural region, after adjustment for age, gender, body mass index, income, blood glucose concentration, diastolic blood pressure and smoking. It is in agreement with the general increase in the prevalence of dyslipidemia in China with increasing urbanization and change in lifestyle. Correspondingly, in the regions with fast economic growth, such as Guangzhou and Shanghai, the mean level of serum cholesterol previously increased markedly [14], [24]. To mention an example, over a 10-year period the serum total cholesterol level for men and women in Guangzhou increased 13.9% and 21.5% in urban areas and 18.9% and 24.9% in rural areas, respectively [14].

The prevalence of dyslipidemia as found in our study (total dyslipidemia: 56.1±0.9%; dyslipidemia without taking into account history of dyslipidemia: 45.1±0.9%) and the prevalence of hypercholesterolemia (19.0±0.7%), hypertrigylceridemia (35.5±0.9%) and hypo HDL-cholesterol (1.7±0.2%) was partially comparable to figures reported in previous investigations. To cite an example, in the InterAsia Collaborative Study, a cross-sectional survey in a nationally representative sample of 15,540 Chinese adults 35 to 74 years of age, 23.8% of the subjects had borderline high total cholesterol (200 to 239 mg/dL), and 9.0% had high total cholesterol (≥240 mg/dL). The population estimates for borderline high (130 to 159 mg/dL), high (160 to 189 mg/dL), and very high ≥190 mg/dL) LDL- cholesterol were 17.0%, 5.1%, and 2.7%, respectively. In addition, 19.2%, had a low HDL-cholesterol (<40 mg/dL). In a recent study on 19,003 suburban Beijing residents aged 18 to 76 years, the age-standardized prevalence of dyslipidemia was 30.3% [26].

Our findings may have public health implications. Traditionally, mortality from coronary heart disease in China was infrequent and was estimated to be only 10% of that in Western populations [22]. A low serum total cholesterol level related to a low habitual dietary intake of fat and cholesterol was considered to be the main underlying reason for the low coronary heart disease mortality in China [23]. In the recent InterASIA study [15], a relatively high mean level of serum cholesterol but a low rate of hypercholesterolemia control was noted. These findings were confirmed by our study which was conducted 5 years after the InterASIA study. It might explain the recent rapid increase in coronary heart disease mortality in China. Furthermore, the findings from the InterASIA study as well as our results suggest that without a national emphasis on prevention, treatment, and control of dyslipidemia, the societal burden of cardiovascular diseases in China will continue to increase in the near future.

Factors associated with dyslipidemia in our study were higher age, female gender, urban region, higher body mass index, income, blood concentration of glucose and diastolic blood pressure, and smoking. In contrast, the level of education, systolic blood pressure and alcohol consumption were not related with dyslipidemia. The association between dyslipidemia and higher age agrees with all previous population-based and hospital-based studies on the same topic [2], [5]–[23]. In a similar manner, the previous studies as our study reported on relationships between dyslipidemia and female gender, urban region, higher body mass index, income, blood concentration of glucose and diastolic blood pressure, and smoking. It may suggest that the factors leading to dyslipidemia and the consequences of dyslipidemia (such as increased body mass index) are similar across ethnic borders. It may imply that, as already pointed out above, the increasing rate of dyslipidemia in China may lead to similar cardiovascular and cerebrovascular consequences as it did in Western countries several decades ago.

The rate of awareness among the subjects with dyslipidemia in the present investigation was 50.9%, which was unexpectedly high. In previous studies from China such as the InterASIA study, the awareness rate was less than 10%. The reason for the unexpectedly high awareness rate may be differences in the regions included into the studies. The average income in the urban regions included into the Beijing Study was considerably higher than in other urban regions of Beijing (1688 RMB (Yuan) versus 866 RMB). In addition, the health care system in the urban study regions which also included quarters with government employees was better developed than in other urban regions of Beijing. In parts of the urban study regions, there was a relatively high standard with some communities supplying free health care examinations, and in these areas, the cost for medical care was covered by the government. Correspondingly, the rate of awareness of dyslipidemia was significantly associated with living in the urban region. Without doubt, the relatively high income level and the relatively well developed health care system in the urban regions of the present study may have artificially increased the awareness rate of dyslipidemia in our study. In a similar manner, the rural regions included in our study had a better developed health care system than other rural regions in the vicinity so that also the data on the awareness of dyslipidemia from the rural regions may have a bias and may be artificially high. In addition, the first survey performed in 2001 included a questionnaire with questions on the presence, duration and treatment of dyslipidemia. Although the blood lipids were not measured in 2001, the questionnaire might have made the study participants aware of the risks of dyslipidemia, so that they got their blood examined between 2001 and 2006. It may have led to a bias with an artificially high rate of awareness of dyslipidemia in the survey of 2006. The data on the awareness of dyslipidemia as found in the present study may, therefore, not be representative for the whole country.

Potential limitations of our study should be mentioned. First, a major concern in any prevalence study is non-participation. In the present study, out of 5324 eligible individuals, 4439 subjects participated in the examination in the year 2001. Out of these 4439 subjects, 3251 (73.2%) returned for the follow-up examination in the year 2006 in which the blood pressure measurements were performed. Based on the originally eligible number of 5324 individuals from the year 2001, the number of 3251 participants in the survey of 2006 indicates a participation rate of 61.1%. The participants of the survey 2006 compared with the non-participants of the survey 2006 were significantly younger, came more often from the rural region, and had a higher level of education. They did not, however, vary in significantly income and gender. One may, therefore, infer that the participation rate may still be acceptable to draw conclusions about the prevalence and associated factors of dyslipidemia in the Greater Beijing area. Second, another limitation of the study may be the question how representative the rural region and the urban regions of Greater Beijing are for the remaining provinces of China. The unexpectedly high rate of awareness of dyslipidemia particularly in the urban region may suggest that one may be cautious in transferring the data of awareness of the diseases to other less developed regions in China. This may, however, not account for the prevalence of dyslipidemia and its associated factors.

In conclusion, dyslipidemia was present in about 56% of the study population (aged 45+ years) in Greater Beijing. Factors, which were likely associated with dyslipidemia, were higher age, female gender, urban region, higher body mass index, higher income, higher blood concentration of glucose, higher diastolic blood pressure, and smoking. In the examined study population, the treatment rate of dyslipidemia was 24% with about 60% of the treated subjects still having uncontrolled dyslipidemia.

## Methods

### Study Participants

The Medical Ethics Committee of the Beijing Tongren Hospital approved the study protocol and all participants gave written informed consent. The eligibility criterion for the study was an age of 40 or more years. The Beijing Eye Study is a population-based study performed in the region of Greater Beijing. The study was carried out in 7 communities. Three of the communities were located in the Daxing District in the village area of Yufa situated south of Beijing area about 50 to 100 km from the center of Beijing. Four communities were located in the Haidian urban district of the Northern part of Central Beijing. The reason to perform the study in a rural area and in an urban area was that both areas differed markedly in the level of education, access to medical care, mobility, frequency of hereditable diseases, and way of life. In the rural areas, health care services and a referral system to ophthalmologists were often not available, and the cost for medical care was usually not covered by the government. In the urban areas, health care was at a relatively high standard with some communities supplying free ophthalmic examinations, and in these areas, the cost for medical care were covered by the government.

All people residing in the communities were officially registered by name, gender and age at the local mayor's office. Using this register as the sampling frame, all subjects living in the 7 communities and fulfilling the inclusion criterion of an age of 40+ years were eligible for the study. Home visits were performed according to the registration list, and the eligibility criteria for the study, an age of 40 or more years, was confirmed by door-to-door enrollment. Out of 5324 eligible individuals, 4439 individuals (2505 women) participated (response rate: 83.4%). The mean age was 56.2±10.6 years (range: 40–101 years). The study was described in detail recently [27]–[29]. To assess the public health impact and to address how representative the sample of the study was for the total population of China, the demographic data of the study population was compared with census data available for the population of China and of Beijing [30]. The average monthly income per capita in the rural areas of Beijing was similar in the .census (391 RMB (Yuan)) and in the rural part of the study population (393 RMB (Yuan)). The income figures of the urban regions were higher in the Beijing Eye Study population (1688 RMB (Yuan)) than in the census (866 RMB (Yuan)). We repeated the study in the year 2006 by re-inviting all participants from the previous survey, and 3251 subjects (1838 (56.5%) women) participated (response rate: 73.2%; 1500 (46.1%) subjects from the rural region). The mean age was 60.4±10.0 years (range: 45–89 years). For the present study, only data measured in the survey of 2006 were taken, and only subjects with blood pressure measurements were considered.

### Methods

All examinations were carried out in the communities, either in schoolhouses or in community houses. Trained research technicians asked the study participants questions from a standard questionnaire providing information on demographic variables such as age, gender, level of education, occupation, family income, known diagnosis of dyslipidemia, and current treatment of dyslipidemia. In the year 2006, blood samples were additionally taken. The serum concentrations of total cholesterol, triglycerides, high-density lipoprotein-cholesterol (HDL-C) and low-density lipoprotein-cholesterol (LDL-C) were measured using the enzymatic method with a Hitachi 7600 auto-analyzer (Hitachi, Tokyo, Japan). Reagents of the same batch were used. Dyslipidemia was defined as any of hypercholesterolemia (total cholesterol concentration ≥5.72 mmol/L (220 mg/dL)) or hypertriglyceridemia (triglyceride concentration ≥1.70 mmol/L (150 mg/dL)) or low high-density lipoprotein-cholesterol (HDL-C concentration ≤0.91 mmol/L (35 mg/dL)) [31]. Total prevalence of dyslipidemia was defined dyslipidemia and / or a positive history for dyslipidemia. For the statistical analyses, the total prevalence of dyslipidemia (abnormal blood examinations results and / or positive history for dyslipidemia) was taken.

### Body weight and height and the arterial blood pressure were also measured

Awareness of dyslipidemia was defined as self-report of any previous diagnosis of dyslipidemia by a healthcare professional. Treatment of dyslipidemia was defined as the use of a pharmacological treatment to lower blood lipids during the previous 2 weeks. Participants were considered to have controlled dyslipidemia if their total cholesterol concentration was 5.2 mmol/l (<200 mg/dL), or to have controlled LDL cholesterol concentration if their LDL cholesterol was <3.38 mmol/L (130 mg/mL) [15].

### Statistical Analysis

Inclusion criterion for the present study was the availability of serum measurements of total cholesterol, triglycerides, and HDL-C measurements. The statistical analysis was performed using a commercially available statistical software package (SPSS for Windows, version 17.0, SPSS, Chicago, IL). The data were given as mean ± standard deviation. Logistic regression was used to investigate the associations of the binary dependent variable “presence of dyslipidemia” with the continuous or categorical independent variables, such as age, gender, area, body mass index, the concentrations of glucose, blood pressure, education and income. Confidence intervals were presented. All *P*-values were two-sided and were considered statistically significant when the *P*-values were less than 0.05. Based on the data of the 2000 China population census, the mean data and the prevalences were adjusted to calculate the total number of subjects with dyslipidemia in China [32].

## References

[1] Murray CJL, Lopez AD (1997). Mortality by cause for eight regions of the world: Global Burden of Disease Study.. Lancet.

[2] Wu Z, Yao C, Zhao D, Wu G, Wang W (2001). Sino-MONICA project: a collaborative study on trends and determinants in cardiovascular diseases in China, Part I: morbidity and mortality monitoring.. Circulation.

[3] Popkin BM, Horton S, Kim S, Mahal A, Shuigao J (2001). Trends in diet, nutritional status, and diet-related noncommunicable diseases in China and India: the economic costs of the nutrition transition.. Nutr Rev.

[4] Yusuf S, Reddy S, Ounpuu S, Anand S (2001). Global burden of cardiovascular diseases: part I: general considerations, the epidemiologic transition, risk factors, and impact of urbanization.. Circulation.

[5] Gordon DJ, Probstfield JL, Garrison RJ, Neaton JD, Castelli WP (1989). High-density lipoprotein cholesterol cardiovascular disease. Four prospective American studies.. Circulation.

[6] Assmann G, Schulte H, von Eckardstein A, Huang Y (1996). High-density lipoprotein cholesterol as a predictor of coronary heart disease risk. The PROCAM experience and pathophysiological implications for reverse cholesterol transport.. Atherosclerosis.

[7] Goldbourt U, Yaari S, Medalie JH (1997). Isolated low HDL cholesterol as a risk factor for coronary heart disease mortality. A 21-year follow-up of 8000 men.. Arterioscler Thromb Vasc Biol.

[8] Stamler J, Daviglus ML, Garside DB, Dyer AR, Greenland P (2000). Relationship of baseline serum cholesterol levels in 3 large cohorts of younger men to long-term coronary, cardiovascular, and all-cause mortality and to longevity.. JAMA.

[9] Blood pressure, cholesterol, and stroke in eastern Asia. Eastern Stroke and Coronary Heart Disease Collaborative Research Group (1998). Lancet.

[10] Verschuren WM, Boerma GJ, Kromhout D (1994). Total and HDL-cholesterol in The Netherlands: 1987–1992. Levels and changes over time in relation to age, gender and educational level.. Int J Epidemiol.

[11] Wu ZS, Yao CH, Chen DY, Li N, Zhang M (1988). The Sino-MONICA-Beijing Study: report on results between 1984 and 1986.. Acta Med Scand Suppl.

[12] People's Republic of China–United States Cardiovascular and Cardiopulmonary Epidemiology Research Group (1992). An epidemiological study of cardiovascular and cardiopulmonary disease risk factors in four populations in the People's Republic of China. Baseline report from the P.R.C.-U.S.A. Collaborative Study.. Circulation.

[13] Yao C (1993). [Sino-MONICA project: comparison of risk factors of cardiovascular diseases in 13 provinces or cities and the trend of their changes].. Zhonghua Liu Xing Bing Xue Za Zhi.

[14] Li YH, Li Y, Davis CE, Tao S, Folsom AR (2002). Serum cholesterol changes from 1983–1984 to 1993–1994 in the People's Republic of China.. Nutr Metab Cardiovasc Dis.

[15] He J, Gu D, Reynolds K, Wu X, Muntner P (2004). Serum total and lipoprotein cholesterol levels and awareness, treatment, and control of hypercholesterolemia in China.. Circulation.

[16] Wu Z, Yao C, Zhao D, Wu G, Wang W (2004). Cardiovascular disease risk factor levels and their relations to CVD rates in China–results of Sino-MONICA project.. Eur J Cardiovasc Prev Rehabil.

[17] Li Z, Yang R, Xu G, Xia T (2005). Serum lipid concentrations and prevalence of dyslipidemia in a large professional population in Beijing.. Clin Chem.

[18] Zhao WH, Zhang J, Zhai Y, You Y, Man QQ (2007). Blood lipid profile and prevalence of dyslipidemia in Chinese adults.. Biomed Environ Sci.

[19] Wu F (2007). [The second multi-center survey of dyslipidemia management in China: goal attainment rate and related factors].. Zhonghua Xin Xue Guan Bing Za Zhi.

[20] Liang LR, Wu YF, Zhao LC, Chen Z, Zhu JR (2009). [Differences in goal attainment in clinical management of dyslipidemia in China evaluated by different guidelines].. Zhonghua Xin Xue Guan Bing Za Zhi.

[21] Kang WM, Zhang JS, Liu XX, Wang MS, Zhao ML (2009). Prevalence of abnormity of blood lipid and associated factors in health examination population in Beijing.. Chin Med Sci J.

[22] Tao SC, Huang ZD, Wu XG, Zhou BF, Xiao ZK (1989). CHD and its risk factors in the People's Republic of China.. Int J Epidemiol.

[23] Johnson CL, Rifkind BM, Sempos CT, Carroll MD, Bachorik PS (1993). Declining serum total cholesterol levels among US adults. The National Health and Nutrition Examination Surveys.. JAMA.

[24] Jia WP, Xiang KS, Chen L, Lu JX, Wu YM (2002). Epidemiological study on obesity and its co-morbidities in urban Chinese older than 20 years of age in Shanghai, China.. Obes Rev.

[25] Zhou B, Rao X, Dennis BH, Li Y, Zhuo Q (1995). The relationship between dietary factors and serum lipids in Chinese urban and rural populations of Beijing and Guangzhou.. Int J Epidemiol.

[26] Zhang L, Qin LQ, Liu AP, Wang PY (2010). Prevalence of risk factors for cardiovascular disease and their associations with diet and physical activity in suburban Beijing, China.. J Epidemiol.

[27] Xu L, Li J, Cui T, Hu A, Fan G (2005). Refractive error in urban and rural adult Chinese in Beijing.. Ophthalmology.

[28] Xu L, Wang S, Wang YX, Wang YS, Jonas JB (2008). Prevalence of arterial hypertension in the adult population in rural and urban China: the Beijing eye study.. Am J Hypertens.

[29] Xu L, Xie X, Wang S, Wang Y, Jonas JB (2008). Prevalence of diabetes mellitus in China.. Exp Clin Endocrinol Diabetes.

[30] Beijing Municipal Statistical Bureau (2001). Beijing Statistical Yearbook 2001..

[31] The groups of preventing dyslipidaemia of Chinese journal of cardiology (1997). Propose of preventing dyslipidaemia.. Chin J Cardiol.

[32] The fifth Census of Chinese population in the year 2000.. http://www.stats.gov.cn.

